# Detection of Rare Antimicrobial Resistance Profiles by Active and Passive Surveillance Approaches

**DOI:** 10.1371/journal.pone.0158515

**Published:** 2016-07-08

**Authors:** Alison E. Mather, Richard Reeve, Dominic J. Mellor, Louise Matthews, Richard J. Reid-Smith, Lucie Dutil, Daniel T. Haydon, Stuart W. J. Reid

**Affiliations:** 1 Boyd Orr Centre for Population and Ecosystem Health, University of Glasgow, Glasgow, United Kingdom; 2 School of Veterinary Medicine, University of Glasgow, Glasgow, United Kingdom; 3 Institute of Biodiversity, Animal Health and Comparative Medicine, University of Glasgow, Glasgow, United Kingdom; 4 Public Health Agency of Canada, Guelph, Ontario, Canada; 5 Department of Population Medicine, University of Guelph, Guelph, Ontario, Canada; 6 Public Health Agency of Canada, St. Hyacinthe, Quebec, Canada; Animal and Plant Health Agency, UNITED KINGDOM

## Abstract

Antimicrobial resistance (AMR) surveillance systems are generally not specifically designed to detect emerging resistances and usually focus primarily on resistance to individual drugs. Evaluating the diversity of resistance, using ecological metrics, allows the assessment of sampling protocols with regard to the detection of rare phenotypes, comprising combinations of resistances. Surveillance data of phenotypic AMR of Canadian poultry *Salmonella* Heidelberg and swine *Salmonella* Typhimurium var. 5- were used to contrast active (representative isolates derived from healthy animals) and passive (diagnostic isolates) surveillance and assess their suitability for detecting emerging resistance patterns. Although in both datasets the prevalences of resistance to individual antimicrobials were not significantly different between the two surveillance systems, analysis of the diversity of entire resistance phenotypes demonstrated that passive surveillance of diagnostic isolates detected more unique phenotypes. Whilst the most appropriate surveillance method will depend on the relevant objectives, under the conditions of this study, passive surveillance of diagnostic isolates was more effective for the detection of rare and therefore potentially emerging resistance phenotypes.

## Introduction

Bacterial infections resistant to antimicrobial drugs pose a great threat to animal and human health [1]. Although many antimicrobials and antimicrobial resistance are natural phenomena and have existed for millennia [2], the widespread use of antimicrobials in animals and humans, and to a lesser extent in plants, has resulted in global selection pressures that have greatly escalated the evolution and spread of resistance [3]. Early detection of antimicrobial resistance (AMR) in bacterial species is critical if we are to understand the drivers of this problem, and moreover, identify and implement candidate mitigation strategies [4–7]. Governments and intergovernmental organisations now recognise the importance of this activity, identifying surveillance for emerging resistance as a priority for infection control [8,9].

Surveillance can be defined in several ways: For the purposes of this analysis and although it may in practice be something different, passive surveillance is defined as the ongoing monitoring of infections, based on diagnostic isolates submitted from clinically diseased individuals or groups; active surveillance is defined as the planned collection of targeted and representative samples, often and this case in surveillance of AMR in the foodchain, from putatively healthy animals. Each approach has different characteristics that affect the nature and strength of inference that can be drawn from the generated data [10,11]. Passive surveillance is usually less costly and more widely used, but may not represent the characteristics of the general host and microbial populations. Biases may also arise through variation in clinician submission behaviours, outbreak episodes, the submission of multiple isolates per individual, and different laboratory methods [12–14]. Furthermore, useful denominator information, such as the number of samples tested but found negative, is frequently not provided. Active surveillance, on the other hand, better reflects the characteristics of the general population, but is usually more costly. In surveillance of AMR in the foodchain, active surveillance of healthy animals, on farm or at slaughter, and retail meat is often used to generate representative estimates for monitoring the trends over time of resistances entering and moving through the foodchain. Detection of the emergence of resistance, despite its importance, is often a secondary objective and may not have been specifically accounted for in programme design. However, in surveillance of AMR in the foodchain, early detection of rare and emerging resistance phenotypes is a critical issue, and identifying which surveillance approach is more efficient equally important in AMR surveillance planning.

Surveillance of AMR has been reviewed previously [4,11–13,15–21], but there has been limited direct comparison of the different systems, particularly using data generated by the same laboratory using the same microbiological techniques. A common approach to comparing data obtained by the two systems is to compare the prevalence of resistance to individual antimicrobials rather than the phenotypes of combined resistances associated with each isolate. Here, using exemplar data from two host species collected by the Canadian Integrated Program for Antimicrobial Resistance Surveillance (CIPARS), we determine and compare the prevalence of resistance to individual antimicrobials of *Salmonella* isolates from active surveillance of healthy animals at slaughter or on farm, and passive surveillance utilising veterinary clinical diagnostic isolates. We contrast the results with an analysis of the diversity of resistance phenotypes observed in isolates from each system, with a focus on assessing the suitability of each method for the identification of new or re-emerging resistances, as determined by the detection of rare phenotypes.

## Materials and Methods

### Data

CIPARS is a national surveillance programme which monitors trends in antimicrobial use and AMR in selected bacteria from animal, human and food sources across Canada [22], including components of both active and passive surveillance.

*Salmonella* Typhimurium var. 5- swine isolates (swine), and *Salmonella* Heidelberg poultry isolates (poultry), were selected as case studies for these analyses. In this analysis, all poultry isolates were from chickens; those from other species, i.e., turkeys and other poultry species, were excluded. Passive surveillance data were generated from diagnostic samples submitted by producers or veterinarians to provincial and private animal health laboratories and then to the *Salmonella* Typing Laboratory at the Laboratory for Foodborne Zoonoses (LFZ), Public Health Agency of Canada (PHAC), Guelph, Ontario for serotyping and/or phage typing. In general, these samples are primarily from diseased animals but may also have been collected from animal feed, the environment of a diseased animal, or non-diseased animals from the same herd or flock; for the purposes of this analysis, isolates obtained from feed, environmental samples or from food products were excluded. Active surveillance data were derived from samples obtained by CIPARS through their on-farm and abattoir components; 25 swine isolates derived from a Canadian food safety surveillance program (FoodNet Canada, formerly C-EnterNet) with an on-farm component similar to that of CIPARS were also included. The CIPARS active surveillance is designed to ensure a random and representative sample of isolates across the year, proportional to the annual slaughter volume (abattoir component) and number of grower-finisher sites (swine on-farm component); further details on the sampling framework are available in the CIPARS annual reports [22]. Isolates included in this analysis were *S*. Typhimurium var. 5- isolates from swine and *S*. Heidelberg isolates from chickens, collected between 2002–2007. Antimicrobial susceptibility testing was performed using the Sensititre^®^ automated microdilution method, and isolates were classified as resistant or non-resistant to each antimicrobial according to breakpoints from the Clinical and Laboratory Standards Institute (CLSI) [23]. Where no CLSI breakpoints were available, minimum inhibitory concentration distributions were used to define epidemiological breakpoints [22]. Full information on 15 antimicrobials was available. Susceptibility testing was conducted by the CIPARS AMR Laboratory, LFZ, PHAC, Guelph, Ontario, Canada. The resistance profile (phenotype) for each isolate was compiled, detailing the antimicrobials to which resistance was demonstrated.

### Prevalence analysis

The prevalence of resistance to each individual antimicrobial and the Wilson score-test-based 95% confidence intervals were calculated [24–26]. To assess whether or not the prevalence of resistance to individual antimicrobials was significantly different between active and passive surveillance isolates, chi-square tests were applied to the numbers of isolates that were susceptible and resistant to each antimicrobial detected by each surveillance system; in cases of low isolate numbers in any cell in the 2x2 table, a Fisher’s Exact test was employed [26]. The significant p-value cut-off was set at 0.0033, Bonferroni-corrected from 0.05 divided to account for the 15 antimicrobials tested.

The percentage resistance was computed to express the average resistance to the 15 antimicrobials of interest among the active and passive surveillance isolates:

$$\text{Percentage Resistance}=\frac{\text{Total}\#\text{Resistances}}{\text{Total}\#\text{Antimicrobials}\times \text{Total}\#\text{Isolates}}$$

where ‘Total # Resistances’ is the summation of all instances of resistance for a given set of isolates to any tested antimicrobial, ‘Total # Antimicrobials’ is the number of antimicrobials against which resistance was assessed, and ‘Total # Isolates’ is the total number of isolates in the given set of isolates. This metric was calculated to compare with previously published results [27,28].

The prevalences of multidrug resistance (MDR), defined as resistance to three or more different antimicrobial classes, in the isolates collected by active and passive surveillance were calculated and compared. The antimicrobial classes examined in this study were quinolones (nalidixic acid and ciprofloxacin), aminoglycosides (kanamycin, gentamicin, amikacin, streptomycin), penicillins (ampicillin, amoxicillin-clavulanic acid), cephalosporins (ceftiofur, ceftriaxone, cefoxitin), tetracyclines (tetracycline), folate pathway inhibitors (sulphonamides, trimethoprim-sulphamethoxazole), and phenicols (chloramphenicol). A similar analysis, where the penicillins and cephalosporins were combined into a single, beta-lactam, drug class, was also performed. Each isolate was classified as either MDR or not MDR, and the numbers of MDR isolates compared between active and passive surveillance using Fisher’s Exact tests.

### Diversity analysis

Two methods were employed to evaluate the diversity of resistance in each dataset, using the AMR phenotype. First, to investigate whether or not the two surveillance methods sampled from a common population of resistance phenotypes, a statistical resampling approach was used, as described in Mather et al. [29]. Briefly, the numbers of phenotypes detected by active and passive surveillance were tabulated. Then, for each of 10,000 iterations, the surveillance method by which each of the isolates in the dataset was sampled was randomly reassigned to another isolate, and the new number of phenotypes in each category (active and passive) tabulated, generating a distribution of results consistent with an origin from a common population. For each dataset separately, the observed numbers of phenotypes obtained by each method were compared to the resampled distributions of the numbers of phenotypes expected, and were considered to be significantly different from that expected if they fell within the first or last 2.5^th^ percentiles of the resampled distributions (p<0.05, two-tailed test). The null hypothesis that resistance phenotypes in isolates obtained by active and passive surveillance derived from a single community of resistance phenotypes was rejected if the observed number of phenotypes was significantly different from that expected for either the active or passive surveillance isolates.

The second method involved calculating ecological measures of diversity for the active and passive surveillance data for each dataset (swine and poultry), as described in Mather et al. [29], using a family of diversity measures related to Rényi’s entropy measures [30], that differentially weight the importance of species richness (the number of total resistance phenotypes) and species abundance (the number of each individual phenotype) (Eq 1). The single parameter *q* (previously α in [29]) determines the extent to which rare resistance phenotypes contribute towards overall diversity.

$$D_{q}\left(p_{1}\cdots p_{s}\right)=\{\;\begin{array}{cc} {\left[\sum_{i=1}^{s}p_{i}^{q}\right]}^{\frac{1}{1-q}} & q\neq 1\\ \prod_{i=1}^{s}p_{i}^{-p_{i}} & q=1 \end{array}$$ (1)

where *p*_1_…*p*_*s*_ are the *s* non-zero relative phenotype abundances

Four specific measures of diversity were examined, Species richness (SR), Shannon entropy (SE), Simpson diversity (SD), and Berger-Parker (BP), as well as across the whole range of the parameter *q*. When *q* = 0 (SR), rare and common phenotypes contribute equally; at *q* = ∞ (BP), rare phenotypes do not contribute to the measure at all, and it is the proportion of isolates that have the most common phenotype that determines the measure. For SE and SD, there is a balance between the contribution of species richness and abundance to the diversity measure. In order to account for differences in sample size, the larger sample of isolates was repeatedly subsampled to the size of the smaller sample of isolates, and diversity measures calculated for the subsamples. For the purposes of our analyses, we were most interested in the results when *q* approached zero, as it is this end of the diversity measure spectrum that relates to new or emergent (rare) phenotypes. The results for all diversity measures for active and passive surveillance isolates were plotted for each dataset.

## Results

There were 463 swine isolates, 51% collected by passive and 49% by active surveillance (S1 Table). There were 41 phenotypes obtained by passive surveillance (17 of which were unique to passive surveillance isolates) and 36 by active surveillance (12 of which were unique to active surveillance isolates); overall, 53 phenotypes (24 common to both surveillance methods) were observed in the swine data. Of the 330 poultry isolates, 25% were collected by passive and 75% by active surveillance (S2 Table). There were 14 phenotypes obtained by passive surveillance (6 of which were unique to passive surveillance isolates), 14 by active surveillance (6 of which were unique to active surveillance isolates), with 20 phenotypes overall (8 common to both surveillance methods) observed in the poultry data.

### Prevalence analysis

The prevalences of resistance to each antimicrobial for passive and active surveillance in the swine and poultry data are presented in Tables 1 and 2. For each antimicrobial, the prevalences of resistance were not significantly different between active and passive surveillance for both the swine and poultry data using the Bonferroni-corrected p-value of 0.0033. The percentages of resistance were similar for the passive and active surveillance isolates for both the swine and poultry data, and are presented in Table 3. In the swine data, 81% (190/236) of the passive surveillance isolates were MDR and 69% (156/227) of the active surveillance isolates were MDR. In the poultry data, 10% (8/84) of the passive surveillance isolates were MDR and 2% (4/246) of the active surveillance isolates were MDR. The prevalence of MDR was higher in passive surveillance isolates for both the swine (p = 0.004) and poultry (p = 0.003) data. Similar results were obtained when the penicillins and cephalosporins were combined into a single beta-lactam drug class.

**Table 1:** Number of resistant isolates (# R) and prevalences of resistance to each antimicrobial with 95% confidence intervals (CI) examined for the isolates obtained by passive and active surveillance of swine *S*. Typhimurium var. 5-. No significant differences were detected by chi-square or Fisher’s Exact tests.

	Active surveillance		Passive surveillance	
Antimicrobial*	# R isolates (total 227)	Prevalence as % (95% CI)	# R isolates (total 236)	Prevalence as % (95% CI)
AMC	4	1.8 (0.7–4.4)	5	2.1 (0.9–4.9)
AP	149	65.6 (59.2–71.5)	180	76.3 (70.5–81.3)
AK	0	0.0 (0.0–1.7)	0	0.0 (0.0–1.6)
GM	4	1.8 (0.7–4.4)	9	3.8 (2.0–7.1)
KA	67	29.5 (24.0–35.8)	77	32.6 (27.0–38.8)
ST	156	68.7 (62.4–74.4)	172	72.9 (66.9–78.2)
CF	0	0.0 (0.0–1.7)	1	0.4 (0.0–2.4)
CX	0	0.0 (0.0–1.7)	0	0.0 (0.0–1.6)
CN	0	0.0 (0.0–1.7)	1	0.4 (0.0–2.4)
NAL	0	0.0 (0.0–1.7)	0	0.0 (0.0–1.6)
CP	0	0.0 (0.0–1.7)	0	0.0 (0.0–1.6)
SX	164	72.2 (66.1–77.7)	198	83.9 (78.7–88.0)
SXT	12	5.3 (3.1–9.0)	29	12.3 (8.7–17.1)
TE	186	81.9 (76.4–86.4)	200	84.7 (79.6–88.8)
CL	121	53.3 (46.8–59.7)	138	58.5 (52.1–64.6)

* AMC: amoxicillin-clavulanic acid; AP: ampicillin; AK: amikacin; GM: gentamicin; KA: kanamycin; ST: streptomycin; CF: ceftiofur; CX: ceftriaxone; CN: cefoxitin; NAL: nalidixic acid; CP: ciprofloxacin; SX: sulphonamides; SXT: trimethoprim-sulphamethoxazole; TE: tetracycline; CL: chloramphenicol

**Table 2:** Number of resistant isolates (# R) and prevalences of resistance to each antimicrobial with 95% confidence intervals (CI) examined for the isolates obtained by passive and active surveillance of poultry *S*. Heidelberg. No significant differences were detected by chi-square or Fisher’s Exact tests.

	Active surveillance		Passive surveillance	
Antimicrobial*	# R isolates (total 246)	Prevalence in % (95% CI)	# R isolates (total 84)	Prevalence in % (95% CI)
AMC	55	22.4 (17.6–28.0)	13	15.5 (9.3–24.7)
AP	99	40.2 (34.3–46.5)	27	32.1 (23.1–42.7)
AK	0	0.0 (0.0–1.5)	0	0.0 (0.0–4.4)
GM	11	4.5 (2.5–7.8)	1	1.2 (0.1–6.4)
KA	4	1.6 (0.6–4.1)	2	2.4 (0.7–8.3)
ST	22	8.9 (6.0–13.2)	13	15.5 (9.3–24.7)
CF	55	22.4 (17.6–28.0)	13	15.5 (9.3–24.7)
CX	0	0.0 (0.0–1.5)	0	0.0 (0.0–4.4)
CN	55	22.4 (17.6–28.0)	13	15.5 (9.3–24.7)
NAL	1	0.4 (0.0–2.3)	0	0.0 (0.0–4.4)
CP	0	0.0 (0.0–1.5)	0	0.0 (0.0–4.4)
SX	13	5.3 (3.1–8.8)	6	7.1 (3.3–14.7)
SXT	1	0.4 (0.0–2.3)	1	1.2 (0.1–6.4)
TE	6	2.4 (1.1–5.2)	5	6.0 (2.6–13.2)
CL	0	0.0 (0.0–1.5)	1	1.2 (0.1–6.4)

* AMC: amoxicillin-clavulanic acid; AP: ampicillin; AK: amikacin; GM: gentamicin; KA: kanamycin; ST: streptomycin; CF: ceftiofur; CX: ceftriaxone; CN: cefoxitin; NAL: nalidixic acid; CP: ciprofloxacin; SX: sulphonamides; SXT: trimethoprim-sulphamethoxazole; TE: tetracycline; CL: chloramphenicol

**Table 3:** Percentages of resistance. Percentages of resistance (% R) for isolates obtained by passive and active surveillance of swine *S*. Typhimurium var. 5- and poultry *S*. Heidelberg, including the % R of passive and active surveillance isolates found by Poppe et al. [27] and Johnson et al. [28] for comparison.

	% R Active Surveillance	% R Passive Surveillance
Swine *S*. Typhimurium var. 5-	25.3	28.5
Poultry *S*. Heidelberg	8.7	7.5
Poppe et al. [27]	6.0	11.8
Johnson et al. [28]	Mean of 11.8% R for active surv. and passive surv. isolates combined, ranging between 0.0–30.9 depending on serovar	

### Diversity analysis

In the poultry data, the number of phenotypes obtained by passive surveillance was significantly higher than expected (in the 98^th^ percentile), and the number of phenotypes obtained by active surveillance significantly lower than expected (in the 2^nd^ percentile). In the swine data, the observed numbers of phenotypes obtained by both passive and active surveillance were within the distribution of expected results (in the 52^nd^ and 5^th^ percentiles of expected results, respectively). Although there is no statistical support for the hypothesis that the phenotypes were sampled from different populations in the swine data, similar trends to those seen in the poultry data were observed, in that the number of phenotypes observed by active surveillance was towards the lower end of that expected.

**Fig 1:**
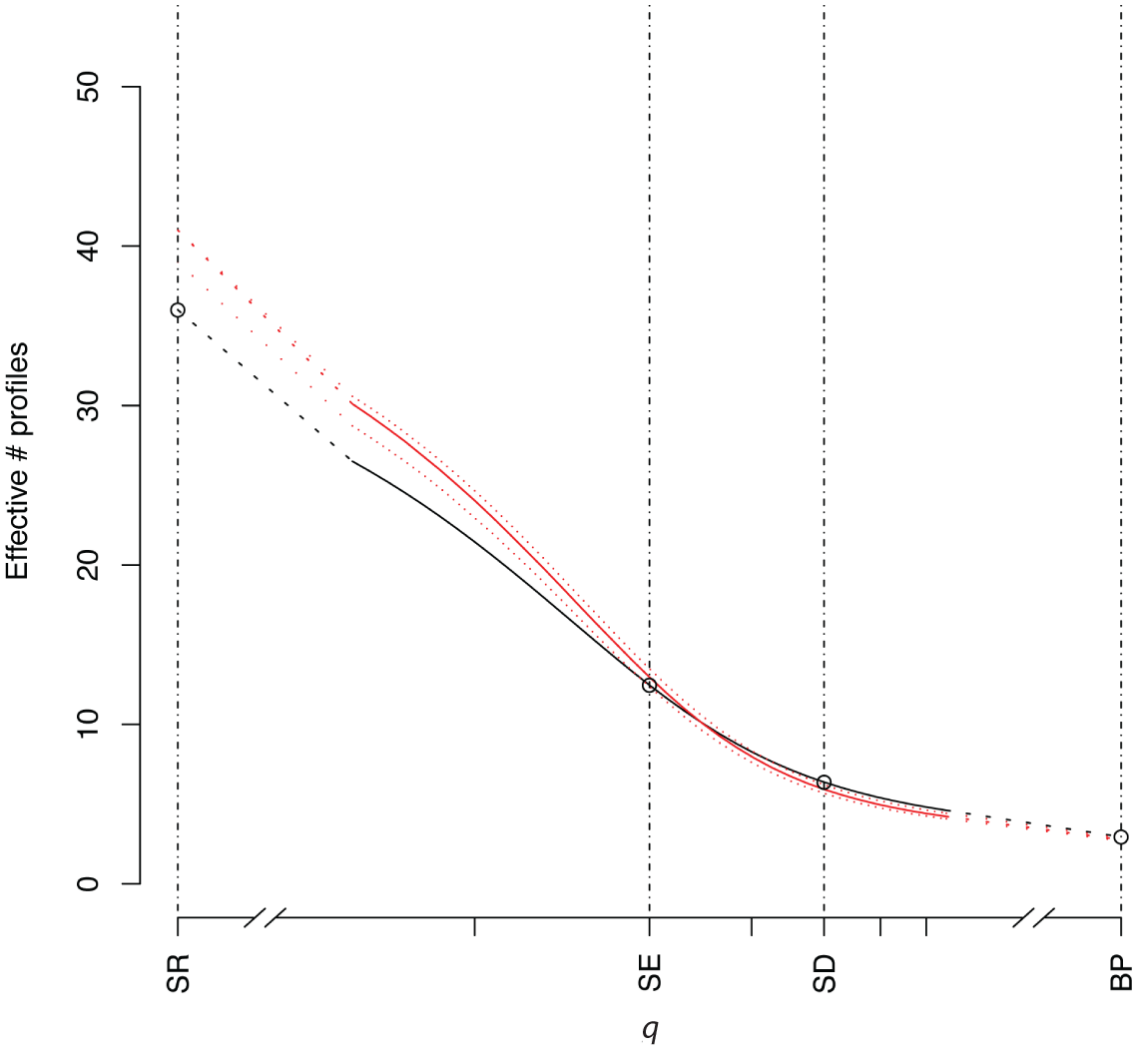
Observed ecological diversities of the swine *S*. Typhimurium var. 5- AMR profiles for all values of the *q* parameter, including Species richness [SR], Shannon entropy [SE], Simpson diversity [SD], Berger-Parker [BP] for passive surveillance (red) and active surveillance (black) isolates with confidence intervals (dotted lines) for the passive surveillance sample generated by subsampling to the size of the active surveillance sample.

**Fig 2:**
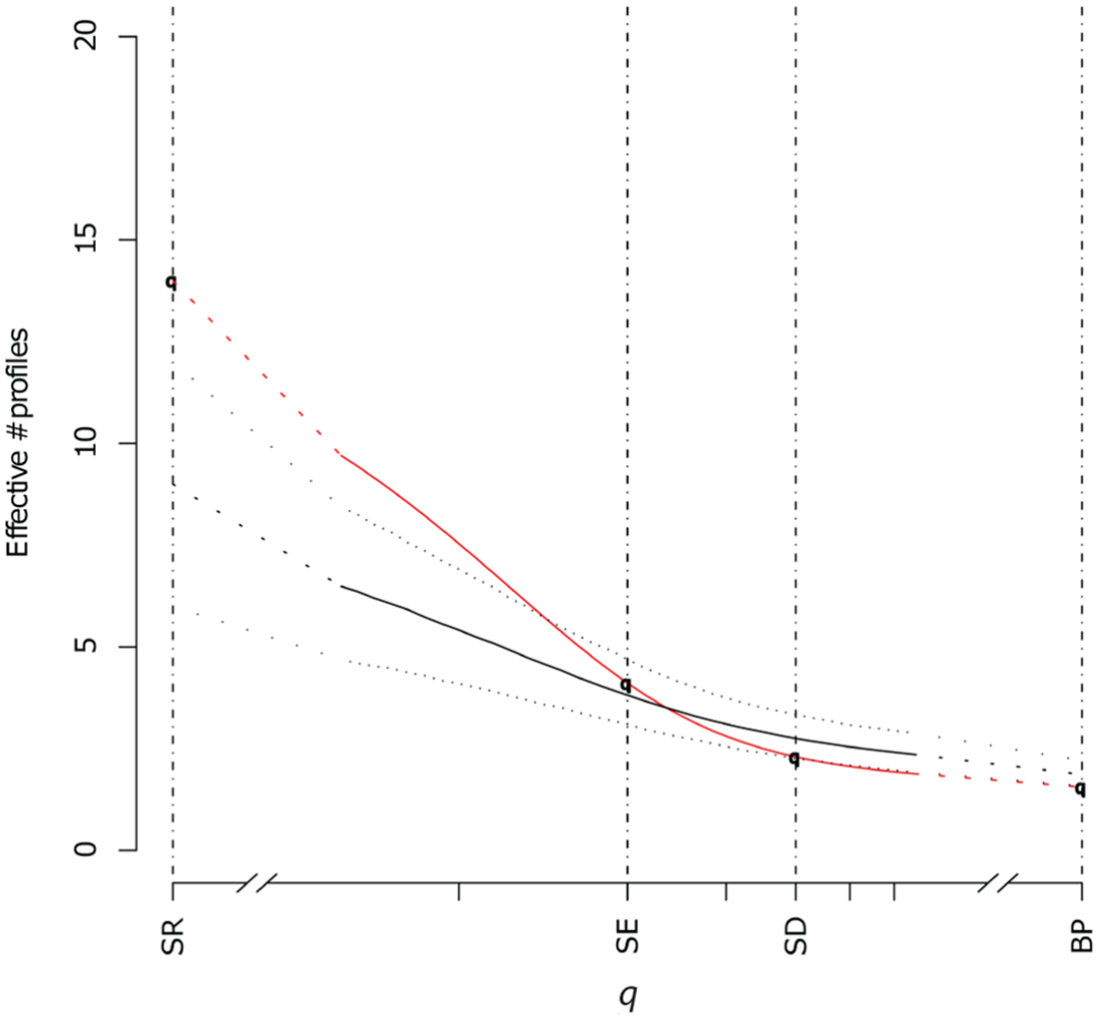
Observed ecological diversities of the poultry *S*. Heidelberg AMR profiles for all values of the *q* parameter, including Species richness [SR], Shannon entropy [SE], Simpson diversity [SD], Berger-Parker [BP] for passive surveillance (red) and active surveillance (black) isolates with confidence intervals (dotted lines) for the active surveillance sample generated by subsampling to the size of the passive surveillance sample.

For several diversity measures in both the swine and poultry data, the diversities of phenotypes obtained by active and passive surveillance were indistinguishable, including those where species richness and abundance both contribute (SE and SD); where rare phenotypes do not contribute at all (BP), the active surveillance phenotypes were slightly more diverse. However, at low values of *q*, where rare phenotypes increasingly contribute to the overall diversity measure, passive surveillance phenotypes were more diverse than active surveillance phenotypes (Figs 1 and 2).

## Discussion

Ongoing surveillance of antimicrobial resistance in bacterial pathogens is central to developing an understanding of the scope of the problem, to monitoring trends, and to evaluating impacts of any intervention strategies. The comparison of active and passive surveillance systems is also important as is the population they are drawing from, as there are differences in terms of the economic and effort costs, as well as the potential biases implicit in each. Sometimes, the two may be used as proxies for each other, but the populations the two systems draw from are different, with the passive approach usually sampling from diseased animal groups, and the active approach typically sampling from healthy animals, although it could be used for diseased animals as well. A passively acquired sample of diseased animals could be a partial subset of an active sample of healthy animals, provided some animals become healthy enough to be re-introduced into the foodchain, but this is not necessarily, nor should it be assumed to be, the case in a given group of animals.

In this analysis we demonstrate that for the prevalence of resistance to individual antimicrobials, the two systems were indeed indistinguishable, using a conservative cut-off. However, when we came to compare the approaches and their ability to detect resistance phenotypes, we demonstrate that passive surveillance using veterinary clinical diagnostic samples was more effective at detecting rare AMR phenotypes of *Salmonella* than active surveillance of healthy animals.

Starting with the individual resistances and examining the prevalence of resistance, a commonly examined measure when investigating AMR, we found no significant difference in the prevalences of resistance to individual antimicrobials or in the overall percentage of resistance between isolates obtained from passive and active surveillance. The evidence from previously published work has been mixed: Johnson et al. [28] found generally no difference between clinical (passive) and monitoring (active) isolates when the animal source of isolates was considered, but Poppe et al. [27] reported that clinical isolates were more likely to exhibit AMR than isolates collected through targeted surveys. Investigations of other diseases and disease causing agents, such as dengue [31], vancomycin-resistant enterococci [32], and invasive pneumococcal disease [33], have shown that passive and active surveillance can result in different prevalence estimates.

However, considering resistance to individual antimicrobials rather than the resistance profile ignores the co-selection of resistance that can occur through gene linkage, and can be misleading if considered alone; using the entire resistance phenotype implicitly takes any such dependencies into account, which is why the phenotype was used as the main unit of analysis for the diversity analyses. When MDR is considered, there was a significantly higher prevalence of MDR in the passive surveillance isolates than in those collected through active surveillance. Furthermore, examining diversity rather than prevalence provides a different perspective with which to compare the two systems. Here we tested whether or not the observed numbers of phenotypes support the hypothesis that the passive and active approaches were sampling from the same bacterial population, granted that the host populations differed in their state of wellness. Using this statistical resampling approach to assess mixing of the sampled bacterial populations, we demonstrated differences between our two examples. In the case of the poultry data, the number of phenotypes detected by passive surveillance of animal clinical isolates was significantly higher than expected, and the number detected by active surveillance of isolates from healthy animals significantly lower than expected, suggesting here that the two approaches may not have been sampling from the same population of phenotypes, or that some bias had occurred at the sampling level for one, or both, of the surveillance methods. In contrast, in the swine data, the numbers of phenotypes detected by passive and active surveillance were as expected, although the results were in the same direction as the poultry data. Whether this difference between livestock species is due to differences in the pathogen, the host or sampling is not clear. There are well-documented differences in the AMR patterns of different *Salmonella* serovars [5,27], and there are inherent differences in the biology of the two animal species and the antimicrobial use, management and husbandry practices in each. Nevertheless, the poultry data alone demonstrate that approaches that involve the entire phenotype rather than the individual resistance can yield additional information and potentially lead to alternative conclusions.

Our findings when we considered only rare phenotypes were more encouraging. Regardless of whether distinguishable or common microbial communities are being sampled, a key issue for early detection is the ability to identify rare phenotypes—newly emergent resistance patterns being rare by definition, although rare phenotypes are not necessarily emergent. For both our datasets, passive surveillance of diseased animals was more successful. At low values of *q*, the passive surveillance isolates had greater diversity than those acquired by active surveillance for both swine and poultry, demonstrating more unique profiles. There are a number of possible biological explanations why this might be the case, including but not limited to bacterial population, husbandry and antimicrobial exposure differences between healthy and clinically ill hosts, as already discussed. Our findings are consistent with those of Afema et al. [34], who examined *Salmonella* from clinical and non-clinical dairy cattle sources, and those of Perron et al. [35], who examined *S*. Typhimurium DT104 isolates from clinically diseased and asymptomatic pigs in a cross-sectional study, where both studies found greater diversity of resistance phenotypes in clinical isolates.

The results of our analyses relate to these particular host/bacterium combinations in Canada; similar assessments in other host species, bacteria, and countries would be useful to determine whether or not the observations made here are consistent in other settings. There are other definitions of passive surveillance, and such surveillance systems in other countries may include other types of sampling, such as repetitive sampling from commercial sources or samples collected for purposes other than AMR investigation, the influence of which would need to be assessed. With respect to the poultry data, there may be isolates from breeding stock and layers as well as broilers included in the passive surveillance sample, whereas the active surveillance isolates were derived from broilers, and this may have led to differences in the two surveillance samples. In addition, while isolates obtained from clinical cases are likely to be from animals of all ages, there may be a higher proportion of isolates from younger animals, as this age group tends to experience more illness from bacterial diseases [36,37]. In the active surveillance sample, the majority of isolates were from animals of approximately the same age within each animal species, that at which the animals are typically sent to slaughter. There are also several acknowledged biases inherent in passive surveillance [12–14], such as variation in clinician submission behaviour; as there is no additional information to assess these biases in our data, it cannot be identified if any of these biases were present. However, it is noteworthy that many of these potential biases, such as multiple isolates submitted per individual, either relate to cases where prevalence estimation, rather than diversity, is the objective, or relate to heterogeneity of laboratory practice in serotyping, phage typing and susceptibility testing, which was not an issue in these analyses.

The role of passive surveillance has been previously acknowledged in the detection of new resistances [15,38], and there has been some discussion of how this might be improved [15,38,39]. The conclusions from these previous studies suggest that key to the identification of emerging resistances are accurate data, a mechanism for recognising unusual resistance patterns, and the ability to discern unusual results whether as the result of laboratory error or a genuinely new pattern.

There are important implications for public health: If novel resistance types can be identified and the epidemiological risks mitigated, then, with appropriate intervention, the efficacy of antimicrobials might be preserved. Early detection is an important component of supporting any prudent use policy, which must also be balanced with the need to provide antimicrobials to animals or humans who require treatment. Phenotype or strain diversity is a component not routinely considered in AMR surveillance systems, either in their design or in their interpretation but our findings suggest that their routine inclusion warrants further investigation.

## Supporting Information

S1 Table Phenotypic antimicrobial resistance profiles of the swine *S*. Typhimurium var. 5- isolates from active and passive surveillance. Amc: amoxicillin-clavulanic acid; Ap: ampicillin; Ak: amikacin; Gm: gentamicin; Ka: kanamycin; St: streptomycin; Cf: ceftiofur; Cx: ceftriaxone; Cn: cefoxitin; Nal: nalidixic acid; Cp: ciprofloxacin; Sx: sulphonamides; Sxt: trimethoprim-sulphamethoxazole; Te: tetracycline; Cl: chloramphenicol.
(PDF)

S2 Table Phenotypic antimicrobial resistance profiles of the poultry *S*. Heidelberg isolates from active and passive surveillance. Amc: amoxicillin-clavulanic acid; Ap: ampicillin; Ak: amikacin; Gm: gentamicin; Ka: kanamycin; St: streptomycin; Cf: ceftiofur; Cx: ceftriaxone; Cn: cefoxitin; Nal: nalidixic acid; Cp: ciprofloxacin; Sx: sulphonamides; Sxt: trimethoprim-sulphamethoxazole; Te: tetracycline; Cl: chloramphenicol.
(PDF)
