# Supplementary material for: Detection of Rare Antimicrobial Resistance Profiles by Active and Passive Surveillance Approaches
Source: PLoS One. 2016 Jul 8;11(7):e0158515. doi: 10.1371/journal.pone.0158515 (PMC4938605; doi:10.1371/journal.pone.0158515)
Supplement: S1 Table — Amc: amoxicillin-clavulanic acid; Ap: ampicillin; Ak: amikacin; Gm: gentamicin; Ka: kanamycin; St: streptomycin; Cf: ceftiofur; Cx: ceftriaxone; Cn: cefoxitin; Nal: nalidixic acid; Cp: ciprofloxacin; Sx: sulphonamides; Sxt: trimethoprim-sulphamethoxazole; Te: tetracycline; Cl: chloramphenicol. (PDF) [file pone.0158515.s001.pdf]

Profile	active/healthy	% active/healthy isolates	passive/clinical	% passive/clinical isolates	phenotype
1	77	33.92	85	36.02	ApClStSxTe
2	2	0.88	5	2.12	ApClSxTe
3	2	0.88	3	1.27	SxTe
4	27	11.89	35	14.83	ApClKaStSxTe
5	4	1.76	2	0.85	KaStSx
6	3	1.32	0	0.00	Ka
7	3	1.32	2	0.85	StSxTe
8	2	0.88	0	0.00	ApKaStTe
9	2	0.88	1	0.42	ApClGmStSxTe
10	1	0.44	0	0.00	ClStSxTe
11	5	2.20	2	0.85	StSx
12	10	4.41	16	6.78	ApKaStSxTe
13	24	10.57	21	8.90	Pansusceptible
14	3	1.32	2	0.85	ApKaSxTe
15	1	0.44	1	0.42	SxTeSxt
16	1	0.44	0	0.00	ApTeSxt
17	3	1.32	2	0.85	ApClStSxTeSxt
18	1	0.44	2	0.85	ApKaStSxTeSxt
19	3	1.32	2	0.85	KaTe
20	23	10.13	7	2.97	Te
21	1	0.44	2	0.85	ApStTe
22	4	1.76	5	2.12	Ap
23	0	0.00	1	0.42	GmSxTe
24	0	0.00	2	0.85	Sx
25	0	0.00	1	0.42	SxSxt
26	0	0.00	2	0.85	GmSxTeSxt
27	0	0.00	1	0.42	ApGmKaStSxTe
28	0	0.00	2	0.85	AmcApClSxTe

29	1	0.44	2	0.85	ApSxTeSxt
30	2	0.88	2	0.85	ApClKaStSxTeSxt
31	0	0.00	1	0.42	AmcApCnCfClStSxTe
32	2	0.88	3	1.27	ApStSxTeSxt
33	0	0.00	2	0.85	KaSxTeSxt
34	1	0.44	4	1.69	ApStSxTe
35	1	0.44	1	0.42	AmcApClKaStSxTe
36	3	1.32	1	0.42	AmcApClStSxTe
37	0	0.00	5	2.12	KaStSxTeSxt
38	0	0.00	1	0.42	ApClKaSxTeSxt
39	0	0.00	1	0.42	ApKaTe
40	0	0.00	2	0.85	ApGmKaStSxTeSxt
41	0	0.00	1	0.42	ApGmSxTeSxt
42	0	0.00	2	0.85	StSxTeSxt
43	0	0.00	2	0.85	ApKaSx
44	0	0.00	1	0.42	ApClGmKaStSxSxt
45	0	0.00	1	0.42	ClSxTe
46	8	3.52	0	0.00	KaStSxTe
47	1	0.44	0	0.00	ApClStTe
48	1	0.44	0	0.00	KaSxTe
49	1	0.44	0	0.00	ApClGmKaStSxTeSxt
50	1	0.44	0	0.00	ApClGmKaStSxTe
51	1	0.44	0	0.00	ApTe
52	1	0.44	0	0.00	ApSxTe
53	1	0.44	0	0.00	ApSx
Total	227		236		
