# Supplementary material for: Detection of Rare Antimicrobial Resistance Profiles by Active and Passive Surveillance Approaches
Source: PLoS One. 2016 Jul 8;11(7):e0158515. doi: 10.1371/journal.pone.0158515 (PMC4938605; doi:10.1371/journal.pone.0158515)
Supplement: S2 Table — Amc: amoxicillin-clavulanic acid; Ap: ampicillin; Ak: amikacin; Gm: gentamicin; Ka: kanamycin; St: streptomycin; Cf: ceftiofur; Cx: ceftriaxone; Cn: cefoxitin; Nal: nalidixic acid; Cp: ciprofloxacin; Sx: sulphonamides; Sxt: trimethoprim-sulphamethoxazole; Te: tetracycline; Cl: chloramphenicol. (PDF) [file pone.0158515.s002.pdf]

Profile	active/healthy	% active/healthy isolates	passive/clinical	% passive/clinical isolates	phenotype
1	133	54.07	54	64.29	Pansusceptible
2	55	22.36	10	11.90	AmcApCnCf
3	1	0.41	1	1.19	Te
5	1	0.41	0	0.00	StTe
7	6	2.44	0	0.00	GmStSx
9	8	3.25	4	4.76	ApSt
10	0	0.00	1	1.19	ApKaStTe
11	3	1.22	1	1.19	KaStTe
12	31	12.60	5	5.95	Ap
14	0	0.00	1	1.19	AmcApCnCfClStSxTe
15	2	0.81	1	1.19	ApGmStSx
18	0	0.00	1	1.19	SxSxt
20	1	0.41	2	2.38	ApStSx
22	0	0.00	1	1.19	AmcApCnCfStSx
23	0	0.00	1	1.19	ApStTe
26	2	0.81	0	0.00	GmSx
27	1	0.41	0	0.00	ApGmStSxTe
28	1	0.41	0	0.00	KaSxSxt
34	0	0.00	1	1.19	AmcApCnCfSt
36	1	0.41	0	0.00	ApNal
Total	246		84		
